# Sella turcica variations in patients with transverse skeletal discrepancies versus patients with normal transverse relationships. a cross- sectional study

**DOI:** 10.1186/s12903-023-02988-y

**Published:** 2023-05-17

**Authors:** Nouran Seifeldin, Ahmed Eltimamy, Nora Al Abbady

**Affiliations:** 1 Department of Orthodontics, Faculty of Dentistry, Cairo University, Giza, Egypt; 2 Department of Radiology, Faculty of Dentistry, Cairo University, Giza, Egypt

**Keywords:** Sella turcica, Transverse maxillary deficiency, Bridging

## Abstract

**Background:**

The sella turcica (ST) is a crucial landmark in orthodontics. It is utilized as a reliable predictor of future growth of the skeletal pattern, assisting in early diagnosis and promoting better treatment planning options. The goal of this research was to compare the morphology and bridging of the sella turcica in transverse maxillary deficient malocclusions and malocclusions with normal transverse relationships.

**Methods:**

A total of 52 cone beam computed tomographic (CBCT) images were selected with an age range of 18–30 years. Group I comprised 26 patients previously diagnosed with transverse maxillary deficiency, while group II comprised 26 patients with normal transverse skeletal relationships. The length, depth and diameter of the ST were measured by two observers, the shape was evaluated as round, oval or flat and sellar bridging was calculated in each case. An Independent t-test was used to compare between the sellar dimensions in both groups. For assessment of bridging percentage Chi square test was used.

**Results:**

The mean values of the length, depth and diameter of the sella in group I was 11.09 mm, 8.56 and 12.81 mm respectively and 10.34 mm, 8.24 and 12.38 mm in group II respectively (P ≤ 0.05). No significant differences were found between both groups in any of the sellar dimensions. The rounded ST shape was the most prevalent among both groups (59.6%). Partial ST bridging was found in only 7.7% of group I (p < 0.0001*). Complete ST bridging wasn’t detected in either of the groups.

**Conclusions:**

There was no correlation found between transverse maxillary deficiency and the morphology and bridging of the sella turcica.

## Background

The morphology and dimensions of the sella turcica (ST) and its relation to various malocclusions and syndromes have always been an area of discussion [1, 2]. ST is clearly seen on lateral cephalograms which are routinely requested for orthodontic diagnosis and treatment planning. The ST, or the pituitary fossa, is a saddle shaped bony depression found in the center of the sphenoid bone that houses the pituitary gland. It is bounded by anterior and posterior walls of different embryonic origins. Growth hormone is secreted from the anterior lobe of the pituitary gland. It is known that the growth hormone greatly affects the craniofacial growth through stimulating endochondral ossification and sutural growth [3]. Endochondral bone formation plays a major role in the growth of the cranial base. The sphenoethmoidal, interethmoidal synchondroses and the nasal cartilage are responsible for the transverse, anterior and vertical growth of the maxilla early in life till the age of 6 years. Studies have found that the younger the children at the start of growth hormone treatment (if needed), the greater the residual growth potential and the greater the effect on craniofacial growth [4, 5].

The posterior wall or the dorsum sellae develops from the para-axial mesoderm which is greatly influenced by the notochord. The anterior wall or the tuberculum sellae develops from the neural crest cells [6, 7]. It was found that anomalies or malformations of the posterior wall were associated with brain alterations while malformations of the anterior wall were associated with abnormalities in the frontonasal and maxillary regions [6, 8]. Due to its close correlation to the pituitary gland, variations in the size of the ST may reflect the presence of hormonal imbalance as hyperprolactinemia [9], pituitary adenoma [10] and Williams syndrome [11]. When the size of ST was compared to different sagittal skeletal patterns, a larger size was seen in skeletal Class III subjects while smaller diameter was present in Class II subjects [12]. The diameter and depth of the ST were found to be similar among class I, class II division 1 and class II division 2 subjects yet class II division 2 subjects showed a smaller ST length [13].

Two anterior and two posterior clinoid processes enclose the pituitary fossa. Variations in morphology in the form of fusion of these processes and ossification in the interclinoid ligament can be seen in 3–13% ^2^ of normal populations and is known as sella turcica bridging (STB). Higher incidence of STB was reported in Class III skeletal patterns ^[[1], [14–17]]^, dental anomalies [17], transpositions [18] and canine impaction cases [19].

Unfortunately, 3 dimensional (3D) studies tackling the ST morphology among different genders, races and malocclusions are scarce in the literature and instead, most of our knowledge is derived from measurements performed on 2 dimensional lateral cephalometric measurements. Nevertheless, information correlating transverse malocclusions and ST morphology is unclear. Therefore, the aim of this study was to assess and compare the morphology and incidence of bridging of the ST 3- dimensionally in patients with skeletal transverse maxillary deficiency and orthodontic patients with normal transverse relationships.

## Methods

In this retrospective cross-sectional study, the sample consisted of 52 cone beam computed tomographic (CBCT) scans of patients who received their treatment in the Department of Orthodontics, Faculty of Oral and Dental Medicine, Cairo University. The study comprised two groups, group I included CBCT images of 26 adult patients (6 males, 20 females) previously diagnosed with a skeletal maxillary constriction. All the samples in group I showed a maxilla-mandibular differential index of 5 mm or more [20]. Group II comprised CBCT images of 26 orthodontic patients (8 males, 18 females) with normal transverse skeletal relationships. For both groups, the inclusion criteria were: medically free adults (age range 18–30 years) of both sexes, Skeletal class 1 (ANB angle = 0–4^0^), have no dental anomalies or impactions, non- syndromic, with no facial deformities and not subjected to previous head traumas or surgeries (Table 1). The study was approved by the Research Ethics Committee of the Faculty of Oral and Dental Medicine, Cairo University (reference number: 35-9-22).

**Table 1:** Mean and standard deviation of some baseline measurements in both groups

	SNA		SNB		ANB		Wits Appraisal		Gonial Angle *(Ar-Go-Me)*		Mandibular Plane Angle *(GoGn/ SN)*	
	Mean	SD	Mean	SD	Mean	SD	Mean	SD	Mean	SD	Mean	SD
Group I	81.44	3.69	79.72	3.34	1.88	2.06	-0.97	1.38	129.49	5.32	36.94	3.72
Group II	82.02	1.97	79.67	1.84	2.26	1.31	-1.22	1.44	126.6	8.75	35.11	3.85

### CBCT Imaging

**Fig. 1:**
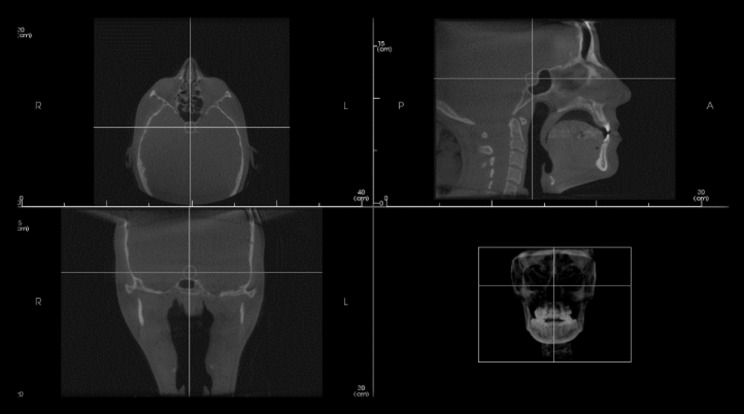
Reorientation of axial, sagittal and coronal planes with centralization of sella turcica in the 3 planes

All the CBCT scans collected in this study were obtained from I-CAT cone beam 3D Imaging unit, with a cylinder field of view of 13 cm high and 16 cm in diameter. The CBCT scans was operated at 120 kVp, milliamperage varied between 5 and 7 mA, for 40 s, voxel size of 0.4 mm and a focal spot of 0.5 mm, The grey scale range of the acquired image was 14 bits. Images were then exported to the computer and in-vivo dental Anatomage software version 5.3.1 was used to open each DICOM file. For standardization of all measurements, the axial, coronal and sagittal planes in all CBCT images were reoriented such that the midsagittal plane was perpendicular to the floor. Moreover, the cursor lines in the reconstructed axial, sagittal, and coronal planes were adjusted such that the ST was centralized in all planes (Fig. 1).

**Fig. 2:**
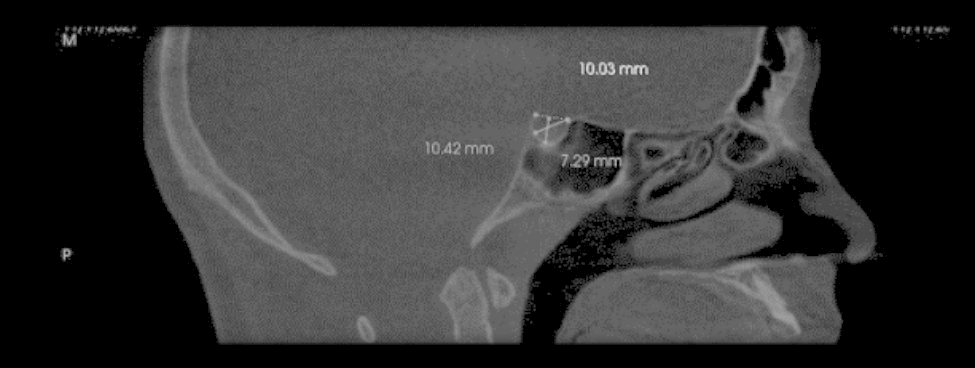
Length, width and diameter measurements of sella turcica

A mid sagittal slice of 0.5 thickness was then selected in all CBCT scans to accurately measure the length, diameter and depth of ST according to Silverman [21] and Kisling [22] (Fig. 2) where:

#### Length

is the horizontal distance between the tip of the dorsum sella to the highest point of tuberculum sella.

#### Width

is the vertical line from the length line to the deepest point on the floor of the ST.

#### Diameter

is an oblique line from the highest point of the tuberculum sella to the deepest point on the inner wall of the ST.

ST bridging was calculated using the Leonardi et al. [17] approach where:

#### Class I

No calcifications, when the length of the ST is greater than three quarters of the diameter.

#### Class II

Partial calcification, when the length of the ST is less than three quarters of the diameter.

#### Class III

complete calcification.

The shape of the ST was defined according to the shape of its floor in the mid sagittal plane as rounded, flat or oval shape [23]. Measurements were recorded by an experienced maxillofacial radiologist (N.A) blinded to both groups. To ensure inter-observer reliability all the measurements were measured once again by an experienced orthodontist (A.E).

### Statistical analysis

Shapiro-Wilk and Kolmogorov-Smirnov test was used to test the normality of the variables’ distribution. All data originated from normal distribution (parametric data) resembling a normal Bell curve.

Sample size calculation [24] revealed that a sample of 20 patients per group was the minimally accepted. When the type I error probability (α) associated with this test was 5% (0.05), the probability (power) was 80% (0.8) with a ratio 1:1 between both groups. Each group finally comprised 26 patients increasing the power of this study to 89% (0.89).

## Results

Despite the mean values of the sellar length, depth and diameter of group I were generally higher than those of group II, no statistically significant differences were detected among the two groups in any of the sellar dimensions measurements (Table 2).

**Table 2:** Minimum, maximum, mean and standard deviation of length, depth and diameter of group I (Transverse maxillary deficiency) and group II (normal) and comparison between them using Independent t-test:

Measurement	Group	Min-Max	M ± SD	Difference between both groups			
				MD ± SED	95% CI		P value
					L	U	
Length	**GI (Transverse)**	9.13–16.28	11.09 ± 1.79	0.75 ± 0.45	-0.16	1.66	0.10
	**G II (Normal)**	7.27–12.98	10.34 ± 1.44				
Depth	**G I (Transverse)**	4.65–11.58	8.56 ± 1.69	0.32 ± 0.41	-0.50	1.14	0.44
	**G II (Normal)**	5.28–10.13	8.24 ± 1.21				
Diameter	**G I (Transverse)**	10.46–15.96	12.81 ± 1.34	0.433 ± 0.38	-0.33	1.20	0.26
	**GII (Normal)**	10.26–15.41	12.38 ± 1.41				

*Min: minimum Max: maximum*

*M: mean SD: standard deviation*

*P: probability level significant at P ≤ 0.05*

For evaluation of the inter-observer reliability of sellar dimensions in both groups, the ICC was calculated and revealed excellent agreement in all the 3 measurements with a range of 0.94 to 0.99 (Table 3).

**Table 3:** Inter-observer reliability evaluation of both groups:

		Inter-observer reliability		
		ICC	95% CI	
			L	U
Group I	Length	0.98	0.932	0.986
	depth	0.99	0.964	0.993
	diameter	0.94	0.871	0.974
Group II	Length	0.96	0.917	0.983
	depth	0.94	0.882	0.976
	diameter	0.95	0.894	0.979

**Fig. 3:**
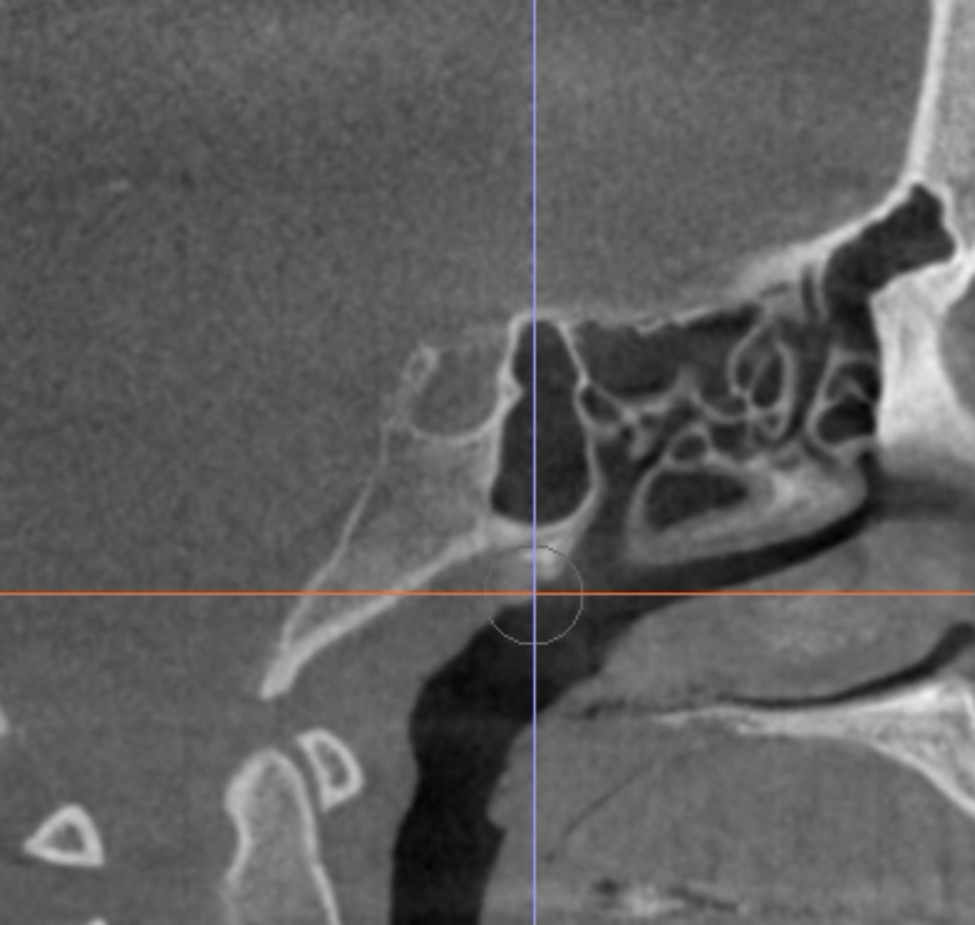
A case showing partial bridging (Class II) of the sella turcica

On correlating both groups to STB, 92.3% of group I patients showed no bridging (class I), 7.7% showed partial bridging (class II), and no cases showed complete bridging (class III) in either of the groups (Fig. 3).

**Fig. 4:**
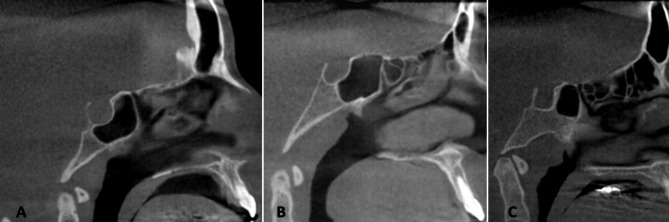
Shapes of sella turcica detected in the samples of both groups; A. round, B.oval, C. flat

**Fig. 5:**
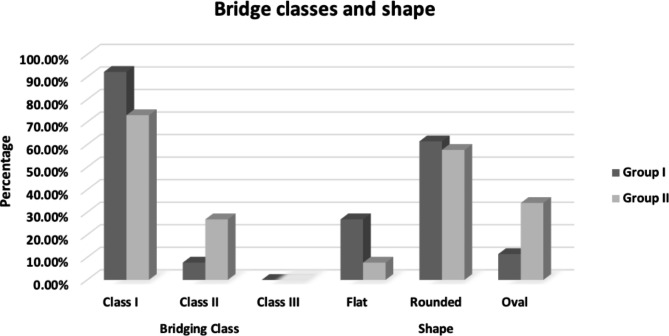
Bar chart showing percentages of different bridging classes and shape in group I & II.

The incidence of class II bridging was higher in group II recording 26.9% (Table 4). Rounded ST shape (Fig. 4) was found to be the highest among the two groups while the flat was the lowest (Table 4). The oval shape was significantly higher in Group II patients recording 34.18% (Fig. 5).

**Table 4:** Frequency and percentages of different bridge classes and shape in group I (transverse) and group II (normal) and comparison between them using Chi square test:

		Group I		Group II		P value
		N	%	N	%	
Bridging Class	Class I	24	92.3%	19	73.1%	0.07
	Class II	2	7.7%	7	26.9%	0.08
	Class III	0	0	0	0	-------
	P value	< 0.0001*		< 0.0001*		
Shape	Flat	7	26.9%	2	7.7%	0.08
	Rounded	16	61.5%	15	57.7%	0.71
	Oval	3	11.5%	9	34.18%	0.02*
	P value	0.006*		< 0.0001*		

*N: count %: percentage*

P: probability level significant at P* ≤ 0.05

## Discussion

Cephalometric radiographs deliver countless diagnostic information to orthodontists before commencing, during and after the orthodontic treatment. The S point is a crucial landmark used in many cephalometric analyses [25, 26]. Moreover, the anterior wall of the ST is considered as one of the important anatomical references used in describing craniofacial patterns [27], superimpositions and growth prediction, taking into account that no further growth changes affect the sella after the age of 15 years [28–30].

Numerous studies have correlated between ST morphology and dental anomalies, craniofacial deviations as well as different skeletal abnormalities in the sagittal plane. Limited studies have correlated between the morphological variations and the above-mentioned deviations 3 dimensionally. Using lateral cephalograms, many studies reported an increase in the STB (whether partial or complete) in cases with palatal canine impactions and teeth transpositions [17], [31–33]. These studies also postulated that the early appearance of STB shouldn’t be overlooked as it may be an indication to future teeth anomalies. Investigating the same correlation using CBCT images, Ortiz et al. [34] negated the presence of any significant association between STB and palatal impacted canines despite reporting the occurrence of STB in 59% in the palatal impacted canine group compared to 50% occurrence in samples with no impactions.

When the shape and size of the ST were evaluated 3- dimensionally in non-syndromic cleft lip and palate patients using CBCT images [23], cleft patients appeared to have a more flattened and shorter sella compared to the non-cleft patients. These findings partially coincide with the results of Alkofide [2] who analyzed the morphology of ST using lateral cephalograms of 95 cleft patients and 190 non- cleft individuals and reported a decrease in its size in cleft subjects. In a similar 2-dimensional study, Alam and Alfawzan [35] also reported a decrease in ST dimension in cleft patients especially those with bilateral cleft lip and palate. Results of the current study revealed that most of the patients in both study groups had a rounded shape ST 59.6%. These findings agree with those of Yasa et al. [23] who reported the rounded shape ST to be the most prevalent (71.8%) among their sample.

In this study, the oval shaped ST was found in only 34.18% patients of group II and yet that was significantly higher than group I.

Lateral cephalograms were also used to correlate morphological changes of ST with different anteroposterior skeletal relationships and the results were controversial. Some studies ^[[9], [36–38]]^ reported an increase in the linear dimensions of the ST in skeletal Class 3 compared to skeletal class 1 and class 2 while other studies [13, 36] found no significant differences in the size of the ST in the three sagittal skeletal variations. This may be due to the different ethnic background of the samples collected in these studies in addition to the unavoidable limitations of the lateral cephalograms as a 2-dimensional diagnostic tool in terms of superimposition and projection errors [40].

When it comes to malocclusions in the transverse dimension, this is the first study to correlate ST morphology and bridging and malocclusion in the transverse plane 3 dimensionally. The current study results showed increased ST linear dimension with mean length, depth and diameter of 11.09 ± 1.79, 8.56 ± 1.69 and 12.81 ± 1.34 mm in patients with transverse maxillary deficiency versus 10.34 ± 1.44, 8.24 ± 1.21, 12.38 ± 1.41 mm in orthodontic patients with normal transverse skeletal relationships with no statistically significant differences between the two groups. Hence, no correlation was found between sellar length, depth or diameter and transverse maxillary deficiency. These results partly resemble those reported by Deniz and Arslan [41] who reported a significant increase in ST length and depth in transverse maxillary deficient patients. In their study, the length and depth of the ST were less than the values reported in the current study (8.91 ± 1.73 and 7.45 ± 1.29 mm in transverse maxillary deficient patients and 8.23 ± 1.97 and 7.24 ± 1.59 mm in normal orthodontic patients). Using CBCT scans in the current study permitted viewing the ST anatomy clearly with no superimpositions of surrounding structures. This in turn aided in assessing the degree of fusion between the anterior and posterior clinoid processes in both groups avoiding interpretation of false or pseudo bridging results.

Different bridging percentages were reported 4.83% ^13^, 9.9% ^17^ and 11.67% ^12^ with results fluctuating between insignificant differences among different skeletal classes [12] and strong correlation of bridging and skeletal class 3 malocclusion [23]. In the midst of this dilemma, Chou et al. [42] reported no significant differences in ST size or bridging percentages measured 3- dimensionally on CBCT images among different sagittal skeletal patterns in a sample of Taiwanese population. Likewise, our results revealed the absence of any correlation between STB and malocclusion in the transverse plane. On the contrary, Deniz and Arslan [41] in their 2-dimensional study on lateral cephalograms reported a higher incidence of bridging in patients with transverse maxillary deficiency. This could be attributed to using lateral cephalograms in assessing the ST morphology and measuring bridging visually (using Axelsson et al. [43] classification) rather than using precise calculations.

In the present study, both genders were included and females comprised the majority of the samples. Yet some studies reported no differences in sellar morphology between adult males and females [2, 12], other studies revealed that gender was significantly correlated with the dimensions and shape of the ST [41, 44]. Hence, results of this study should be interpreted with caution. Besides nourishing the forensic data, results of the current study could share in the diagnostic process and information regarding transverse maxillary deficiencies. Although CBCT images were used, the references that were followed to assess ST morphology and bridging were originally drawn from measurements and norms on lateral cephalograms due to the lack of references established 3 dimensionally. This limitation should be taken into consideration to develop future 3D norms to aid in sorting and classifying the sellar morphology and degree of bridging. Being extracted from Egyptian population data, results of this study shouldn’t be compared to measurements of populations of different ethnic or genetic backgrounds.

## Conclusions

Within the limitations of the current study, no correlation was found between neither sellar size nor shape and transverse maxillary deficiency. Furthermore, sella turcica bridging was not associated with malocclusions in the transverse plane. Occurrence of oval shaped sella turcica was significantly greater in patients with normal transverse relationships.

## Data Availability

The datasets used and/or analysed during the current study are available from the corresponding author ( email: nouran.fouad@dentistry.cu.edu.eg) on reasonable request.

## List of Abbreviations

ST: Sella turcica
CBCT: Cone beam computed tomography
STB: Sella turcica bridging
3D: 3-Dimensional
